# Effectiveness and safety of reduced-port laparoscopic surgery vs conventional multi-port laparoscopic surgery in the treatment of gastric diseases: A meta-analysis

**DOI:** 10.1097/MD.0000000000023941

**Published:** 2021-01-22

**Authors:** Xu Yang, Zhaoting Bu, Maoqin He, Yue Lin, Yuting Jiang, Da Chen, Kaibing Liu, Jun Zhou

**Affiliations:** a Department of Gastrointestinal Surgery, Sun Yat-sen Memorial Hospital, Sun Yat-sen University, Guangzhou, Guangdong Province; b 22 Shuang Yong Road, Graduate School of Guangxi Medical University, Nanning, Guangxi Autonomous Region; c The First Affiliated Hospital, Sun Yat-sen University, Guangzhou, Guangdong Province, China.

**Keywords:** gastric diseases, laparoscopy, meta-analysis, multi-port, reduced-port

## Abstract

This study aimed to compare the effectiveness and safety of reduced-port laparoscopic surgery (RPLS) and conventional multi-port laparoscopic (CMPLS) surgery in the treatment of gastric diseases.

The PubMed, Embase, Cochrane Library, Web of Science, and Chinese Biomedical Literature databases were systematically searched for randomized controlled trials, cohort studies, and case control studies on the use of RPLS vs conventional multi-port laparoscopic surgery in treating gastric diseases from their inception until March 10, 2019. The evaluated outcomes were the operative time, blood loss, length of hospital stay, number of dissected lymph nodes, postoperative complications, and conversions. All of these were compared using Stata software version 12.0.

A total of 18 studies were included, which involved 2938 patients. In studies referring to the comparison between RPLS and CMPLS in treating gastric diseases, the former showed significantly inferior in terms of operative time (*P* = .011) and number of dissected lymph nodes (*P* = .031); but superior results in terms of the estimated blood loss (*P* = .000) and length of hospital stay (*P* = .001) than the latter did; however, the rates of postoperative complications (*P* = .830) and conversions (*P* = .102) were not statistically significant between the 2 groups.

RPLS and CMPLS showed comparable effectiveness and safety in the treatment of gastric diseases in our meta-analysis. Based on the current evidence, we believe that RPLS is an efficacious surgical alternative to CMPLS in the management of gastric diseases because of the shorter hospital stay and reduced blood loss. However, large-scale, well-designed, multicenter studies are needed to further confirm the results of this study.

## 1 Introduction

Laparoscopic surgery has been widely adopted as an alternative to laparotomy in the treatment of gastric diseases. It is reported to be beneficial to patients, due to better early postoperative outcomes, a shorter operative time, and shorter postoperative hospital stay, and a smaller incision compared to that of traditional laparotomy.^[1–3]^ As minimally invasive surgery continues to evolve with time, in keeping with this continued evolution, reduced-port laparoscopic surgery (RPLS) was proposed to further minimize the invasiveness of laparoscopic surgery. Efforts are underway to reduce the invasiveness of laparoscopy, and RPLS is the result of these efforts. Although the research on RPLS is still ongoing because of its limitations in terms of the equipment required and the need for a larger single incision, extensive reports on single-port surgery are found in various clinical and surgical fields.^[4,5]^

RPLS was designed to further minimize wound access trauma by reducing the number of puncture wounds in the abdominal wall. RPLS has been used in various surgeries, such as splenectomy, colectomy, cholecystectomy and gynecological surgeries,^[6–9]^ and its cosmetic merits have been widely accepted.^[6,10,11]^ However, data related to its use in gastric diseases have rarely been reported. Additionally, most studies describing this technique that have been published to date had small sample sizes, rendering them underpowered to assess the safety and feasibility of this surgery,^[12]^ and they often produced controversial or inconclusive results. Current research evaluating the effectiveness and safety of reduced-port vs multi-port laparoscopic surgery in gastric diseases remains lacking.

The aim of this systematic review and meta-analysis was to examine the currently available evidence on the feasibility and safety of RPLS and compare the short-term outcomes after RPLS to those after conventional multi-port laparoscopic surgery (CMPLS).

## 2 Materials and methods

This meta-analysis is based on published data; thus, it is not necessary to seek informed consent from patients.

### 2.1 Literature search

The PubMed, Embase, Cochrane Library, Web of Science, and Chinese Biomedical Medicine (CBM) databases were searched for randomized controlled trials, cohort studies, and case control studies comparing RPLS and CMPLS that were published until March 10, 2019. The following medical subject heading (MeSH) terms were used for the search in all possible combinations: “stomach,” “gastric,” “laparoendoscopic single-site,” “LSS,” “single-port access,” “SPA,” “single-port surgery,” “SPS,” “transumbilical endoscopic surgery,” “TUES,” “laparoendoscopic single-site surgery,” “single-incision laparoscopic surgery,” “SILS,” “transumbilical single port,” “TUSP,” and “single-incision multi-port.” A filter for identifying comparable studies recommended by the Cochrane Collaboration was used to filter out nonrandomized studies in PubMed and Embase. A manual search of the reference lists of relevant articles was also performed. No language or time restrictions were used.

### 2.2 Inclusion and exclusion criteria

The inclusion criteria were as follows:

1. the study design should be a case-match design (randomized controlled trials [RCTs] or controlled clinical trials [CCTs]) comparing RPLS and CMPLS;
2. RPLS performed using laparoscopic or endoscopic instruments, in which case the surgery is referred to as a laparoendoscopic single-site surgery, single-incision endoscopic surgery, reduced-port surgery, or triple-incision laparoscopic surgery;
3. CMPLS referred to as conventional total laparoscopic surgery or conventional laparoscopic-assisted surgery; and
4. studies that included information on at least one of the following outcome measures: the operative time, blood loss, length of hospital stay, number of dissected lymph nodes, postoperative complications, and conversions.

The exclusion criteria were as follows:

1. case reports, quasi-randomized trials, and reviews; and
2. overlapping data.

### 2.3 Data extraction and assessment of the risk of bias

Data were extracted and critically appraised independently by 2 reviewers. The following data were extracted: the first author, publication year, country, study design, interventions, sample size, and outcomes. The risk of bias was also evaluated by the reviewers using the Cochrane Handbook for Systematic Reviews of Interventions.^[13]^ The assessment was based on sequence generation, allocation concealment, blinding, incomplete outcome data, selective outcome reporting, and other sources of bias. Disagreements were resolved by discussion during a consensus meeting with a third reviewer.

### 2.4 Quality assessment of the studies

The PRISMA (Preferred Reporting Items for Systematic Reviews and Meta-Analyses) and AMSTAR (assessing the methodological quality of systematic reviews) guidelines were used. The Newcastle-Ottawa Quality Assessment Scale^[14]^ was used to assess the quality of the studies by 2 independent reviewers. The elements of the scale include the selection of the study groups; the comparability of the groups; and the ascertainment of either the exposure or the outcome of interest. A study was awarded a maximum of 1 star for each numbered item within the selection and outcome categories. A maximum of 2 stars were awarded for comparability. Each study was classified as either poor-quality [0–5] or high quality [6–9], and poor-quality studies were excluded.

### 2.5 Statistical analysis

All data were analyzed using Stata software version 12.0 (Stata-Corp. College Station, TX). The outcome variables that were used for the analysis fulfill the following criteria:

1. means and standard deviations to analyze continuous variables presented in the same scale (ie, the operative time, length of hospital stay, blood loss), and
2. a minimum of 2 studies to analyze identical variables.

Six outcome variables were chosen for the analysis: the operative time (minutes), conversions (n), complications (n), length of hospital stay (days), blood loss (ml), and number of dissected lymph nodes (n). We converted the outcome variables to uniform variables for the ease of analysis. The operative time, blood loss, and number of dissected lymph nodes were used to assess the effectiveness and safety of the operative procedures. The length of hospital stay, conversions, and complications were used to compare the postoperative recovery. The odds ratio and either a random-effects model or fixed-effects model was used to analyze the dichotomous variables according to the presence or absence of heterogeneity. The standardized mean difference was employed to analyze continuous variables. Statistical heterogeneity between studies was evaluated using the Q-based χ^2^ test and the I^2^ statistic, with a *P*-value of less than .05 regarded as statistically significant among the studies. Sensitivity and subgroup analyses were performed to explore the possible explanations for the heterogeneity, and subgroup analyses were used to assess the potential effects of the types of operations being compared: reduced-port laparoscopic distal gastrectomy (RPLDG) vs conventional multi-port laparoscopy-assisted distal gastrectomy (CLADG), reduced-port laparoscopy-assisted total gastrectomy (RP-LATG) vs conventional laparoscopy-assisted total gastrectomy (C-LATG), and single-incision laparoscopic adjustable gastric banding (SILS-AGB) vs laparoscopic multi-port adjustable gastric banding (LAGB). If the number of included studies exceeded 10, the potential publication bias was assessed by visual inspection of the funnel plots based on the primary outcomes. The conclusion indicating “no publication bias” was usually made if the figure was presented with good symmetry.

## 3 Results

**Figure 1:**
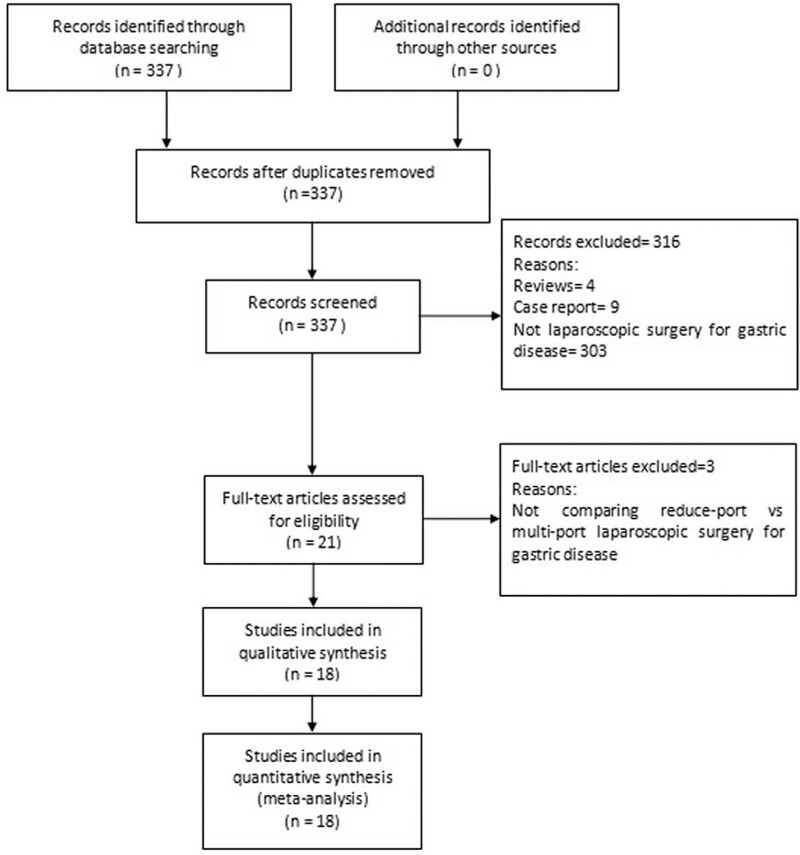
Flow chart for the systematic search and study selection strategy.

Literature selection was conducted using the designed strategy. No randomized controlled trials reporting this subject were found, and 337 relevant citations were identified after removing the duplicates. A total of 316 citations were excluded after reviewing the titles and abstracts. The remaining 21 citations were assessed for eligibility by reviewing the full text. Of these, comparisons of RPLS and CMPLS were considered suitable for the pooled analysis among these citations. After assessing the full-text articles, 3 were excluded. Figure 1 depicts a PRISMA flow chart of the study inclusion and exclusion criteria. 18 studies were eligible for the final meta-analysis.

### 3.1 Characteristics of the included studies

The characteristics of each included study are displayed in Table 1. In total, 2938 patients were randomized to either RPLS (1431 patients) or CMPLS (1507 patients). The articles included in the quantitative synthesis were published between 2009 and 2016. No language restrictions were placed on the search. All the included trials were reported in English. One study included 2 groups data and was divided into 2 groups of comparative data to allow pooled analysis of the outcomes.^[15]^

**Table 1:** Included studies characteristics.

				N				
Study	Study design	Study period	Country	RPLS	CMPLS	RPLS device	CMPLS device	Intervention
RPLDG VS CLADG								
Takeshi Omori^[22]^	CCT	2007–2011	Japan	50	50	Single ports	5 ports	Single-Incision Multi-Port vs Conventional Multi-Port Laparoscopic Distal Gastrectomy
Hideki Kawamura^[29]^	CCT	2010–2011	Japan	30	30	2 ports	5 ports	Reduced-Port vs Conventional Laparoscopy-Assisted Distal Gastrectomy
Sang-Hoon Ahn^[3]^	CCT	2011–2013	Korea	50	50	Single ports	5 ports	Pure Single-Port vs Multi-Port Laparoscopic Distal Gastrectomy
Su Mi Kim^[30]^	CCT	2013–2014	Korea	102	100	3 ports	5 ports	Reduced Port Totally vs Conventional Laparoscopic-Assisted Distal Gastrectomy
Oh Jeong (group 1)^[15]^	CCT	2010–2014	South Korea	49	152	3 ports	5 ports	Duet Laparoscopic vs Conventional totally Laparoscopic Distal Gastrectomy
Oh Jeong (group 2)^[15]^	CCT	2010–2014	South Korea	49	230	3 ports	5 ports	Duet Laparoscopic vs Conventional Laparoscopic-assisted Distal Gastrectomy
Chikara Kunisaki^[23]^	CCT	2010–2011	Japan	20	18	2 ports	5 ports	Reduced-Port vs Conventional Laparoscopic Distal Gastrectomy
Shinsuke Usui^[28]^	CCT	2010–2012	Japan	33	35	3 ports	5 ports	Triple-incision vs Conventional Laparoscopy-Assisted Distal Gastrectomy
RP-LATG VS C-LATG								
Hideki Kawamura^[31]^	CCT	2010–2011	Japan	10	10	2 ports	5 ports	Dual Port vs Conventional Laparoscopy-Assisted Total Gastrectomy
Chikara Kunisaki^[24]^	CCT	2002–2014	Japan	45	45	2 ports	5 ports	Reduced-Port vs Conventional Laparoscopy-Assisted Total Gastrectomy
SILS-AGB VS LAGB								
Ninh T Nguyen^[19]^	CCT	2008–2009	USA	23	23	Single port	5 ports	Laparoendoscopic Single Site vs Conventional Laparoscopic Gastric Banding
ALAN A.SABER^[17]^	CCT	2008–2009	USA	15	12	Single port	5 ports	Single-incision Laparoscopic vs Conventional Multiport Laparoscopic Gastric Banding
Subhashini M.Ayloo^[18]^	CCT	2006–2009	USA	25	121	Single port	5 ports	Laparoendoscopic Single-Site vs Laparoscopic Adjustable Gastric Banding
Sivamainthan Vithiananthan^[25]^	CCT	2007–?	USA	10	20	Single port	4 ports	Single-Incision vs Conventional Laparoscopic Gastric Banding
Matthew Gawart^[27]^	CCT	2009–?	USA	48	50	Single port	5 ports	Laparoendoscopic Single-Site vs Standard Multiport Gastric Bands
Bradley F.Schwack^[20]^	CCT	2008–2010	USA	710	584	Single port	5 ports	Single-Incision vs Non-Single-Incision Laparoscopic Adjustable Gastric Banding
Jennifer Jolley^[21]^	CCT	2011–?	USA	22	37	Single port	5 ports	Single-Incision vs Conventional Laparoscopic Adjustable Gastric Banding
Koji Park^[16]^	CCT	2007–2011	USA	106	100	Single port	5 ports	Laparoendoscopic Single-Site vs Standard Multiport Laparoscopic Adjustable Gastric Banding
Saurav Chakravartty^[26]^	CCT	2009–2010	UK	46	46	Single port	5 ports	Single Incision vs Multiple Incision Laparoscopic Adjustable Gastric Banding

Generally, the included studies showed moderate methodological quality. One case-control study obtained 9 stars.^[16]^ Ten case-control studies received 7 stars^[3,15,17–24]^ because the most important mixed factors were not ideally controlled. Given that the general mixed factors in 2 case-control studies were not controlled, these studies were awarded only 8 stars.^[25,26]^ Five case-control studies received 6 stars.^[27–31]^ No study was excluded based on the Newcastle-Ottawa Quality Assessment Scale.^[14]^

### 3.2 Meta-regression

According to the Cochrane Handbook, when a meta-analysis contains fewer than 10 studies, meta-regression should generally not be considered. Therefore, we simply examined the outcome variables with high heterogeneity, which included more than 10 studies, using a meta-regression model. The analyses indicated that the patient type, body mass index (BMI), gender, and a history of hypogastric operation or any type of abdominal surgery were not significant sources of heterogeneity.

### 3.3 Outcome measurements

#### 3.3.1 Operative time

**Figure 2:**
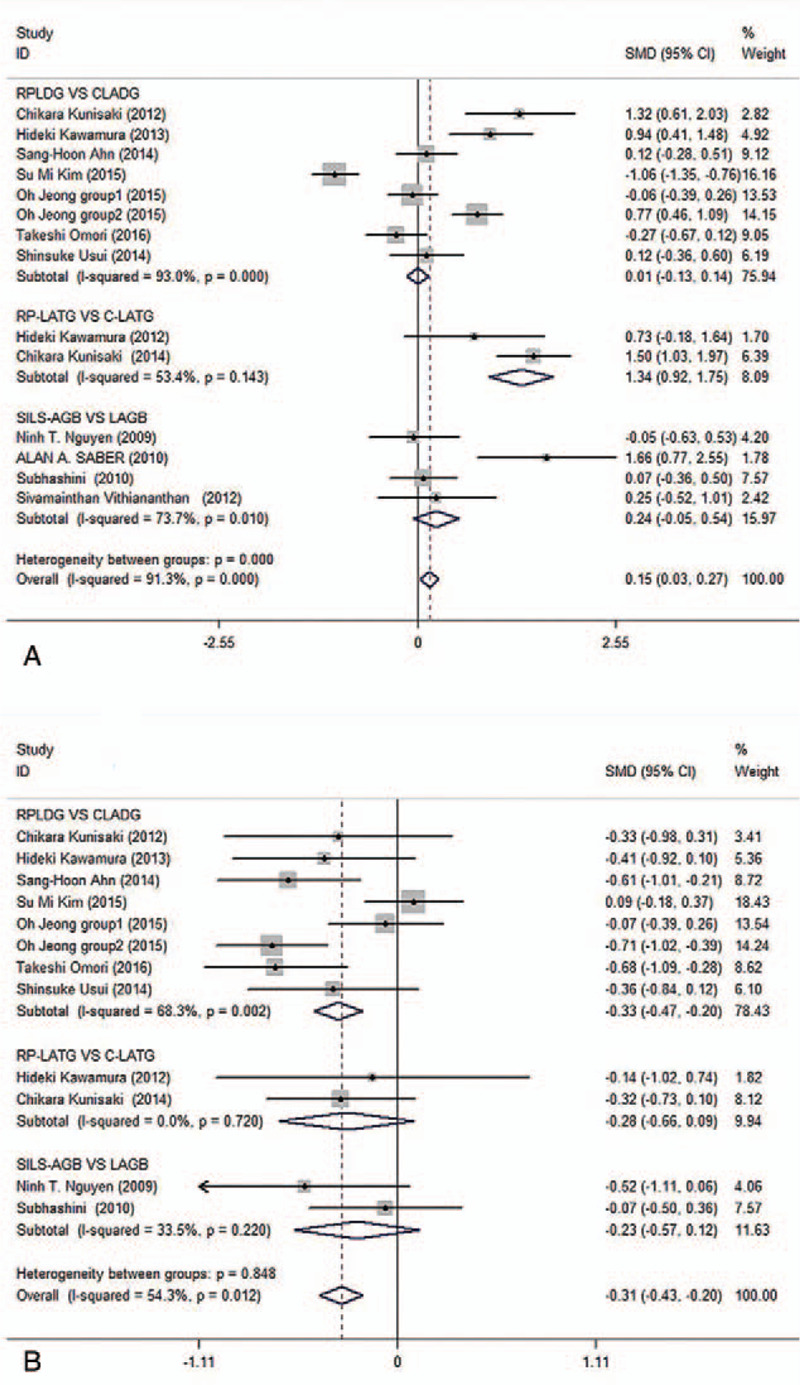
Forest plots of operative time and blood loss in subgroup analysis by ethnicity using a fix-effect model.

Thirteen studies provided data regarding the operative time.^[3,15,17–19,22–25,28–31]^ A total of 1407 patients were included in this meta-analysis, and the subgroup analysis revealed that the operative time was not significantly different between the RPLDG and CLADG subgroups (*P* = .908) or the SILS-AGB and LAGB subgroups (*P* = .108). Conversely, in the RP-LATG vs C-LATG subgroups, the operative time was significantly longer in the RP-LATG group (*P* = .000) than in the C-LATG subgroup, and the overall analysis supported this trend (Fig. 2A) and revealed no publication bias (*P* = .064).

#### 3.3.2 Blood loss

Eleven studies^[3,15,17,19,22–24,28–31]^ provided data regarding the blood loss; they included 1350 patients. The average blood loss was significantly lower in the RPLDG group (*P* = .000) than in the CLADG group. In contrast, there were no significant differences in the quantity of blood loss between the RP-LATG and C-LATG (*P* = .138) or the SILS-AGB and LAGB subgroups (*P* = .200). Based on pooled analysis, there was a significant difference between the RPLS and CMPLS groups in terms of the quantity of blood loss (*P* = .000) (Fig. 2B), which revealed no publication bias (*P* = .479).

#### 3.3.3 Length of hospital stay

**Figure 3:**
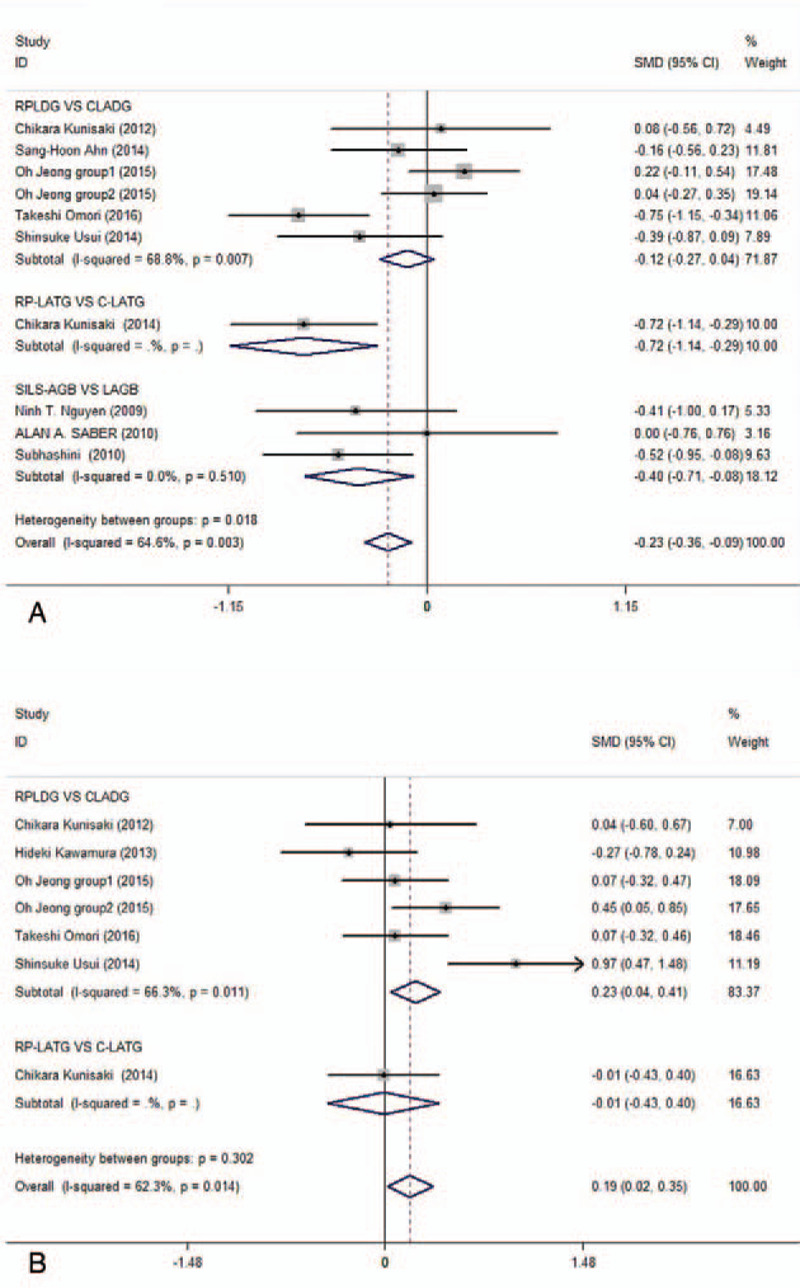
Forest plots of length of hospital stay and number of dissected lymph nodes in subgroup analysis by ethnicity using a fix-effect model.

Nine studies^[3,15,17–19,22–24,28]^ described the length of the hospital stay, and 1098 patients were included in this meta-analysis. The subgroup analysis showed that the length of hospital stay was significantly different among any of the comparative subgroups except the RPLDG and CLADG groups (*P* = .154), and the overall pool estimates supported this trend (Fig. 3A) and showed no publication bias (*P* = .438).

#### 3.3.4 Number of dissected lymph nodes

Early gastric cancer is defined as gastric cancer in which tumor invasion is limited to the mucosa or submucosa, regardless of the presence or absence of lymph node metastases. D2 lymph node dissection is recommended for patients with advanced gastric cancer, whereas for patients with early gastric cancer, less than D2 lymph node dissection is recommended because early gastric cancer rarely metastasizes to the extra-perigastric lymph nodes. The number of dissected lymph nodes was reported by 6 studies^[15,22–24,28,29]^ and 552 patients were included in this meta-analysis. There was no significant difference between the RP-LATG and C-LATG groups (*P* = .950). In contrast, there were significant differences between the RPLDG and CLADG groups (*P* = .017), and the pooled analysis supported this trend (Fig. 3B) and showed no publication bias (*P* = .924).

#### 3.3.5 Postoperative complications

**Figure 4:**
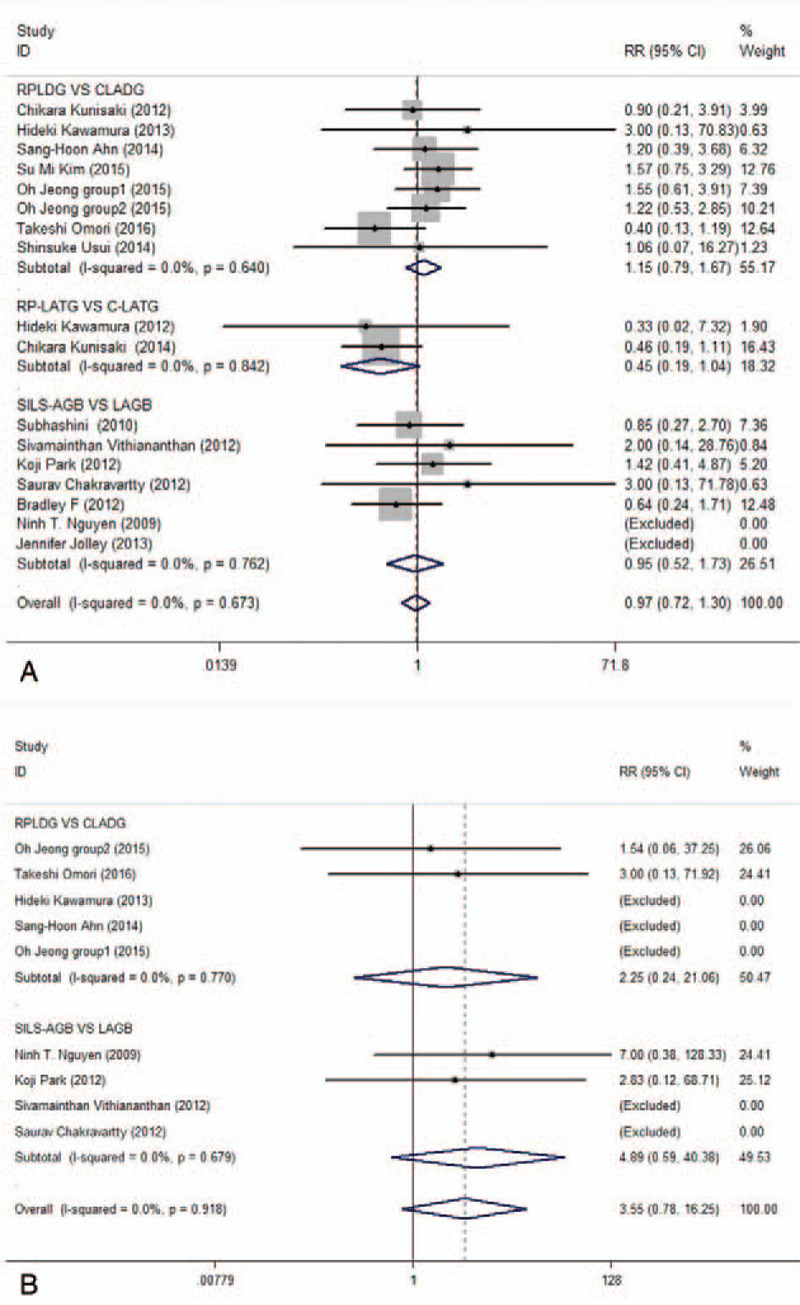
Forest plots of postoperative complications and conversions in subgroup analysis by ethnicity using a fix-effect model.

Complications such as ileus, delayed gastric emptying, intestinal obstruction, anastomotic strictures, anastomotic bleeding, wound-associated complications, stomal obstruction, wound infection, seroma, gastric ulcer, incisional hernia, band slippage, and port dislodgement were observed in this meta-analysis. There were no significant differences in the rates of complications in any comparative subgroup analysis; the pooled analysis also supported this trend across trials (Fig. 4A) and showed no publication bias (*P* = .766).

#### 3.3.6 Conversions

Eight studies^[3,15,16,19,22,25,26,29]^ evaluated conversions, but only 6 patients from 4 studies required conversions. A conversion refers to a switch to open surgery or conventional laparoscopic surgery. The rates of conversions were the same in both treatment subgroups, and the overall estimates also showed no significant difference between the RPLS and CMPLS groups (Fig. 4B) and no publication bias (*P* = .107).

### 3.4 Sensitivity analyses

**Figure 5:**
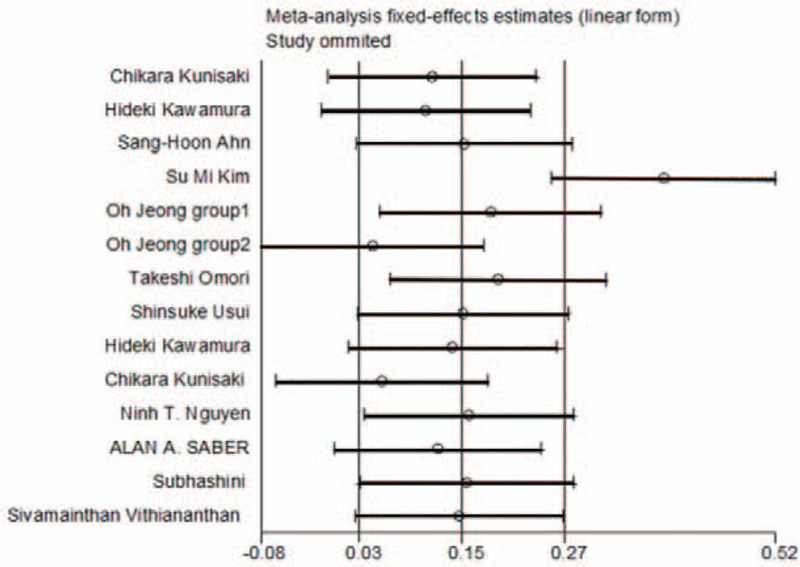
Sensitivity analysis.

The inclusion criteria of this meta-analysis were subjected to a sensitivity analysis to determine whether modifying the inclusion criteria would affect the results. A single study involved in the meta-analysis was deleted each time to reflect the influence of each individual dataset on the pooled standardized mean difference or odds ratio. The corresponding pooled results were essentially unaltered, indicating that our results were statistically sound (Fig. 5).

### 3.5 Risk of publication bias

**Figure 6:**
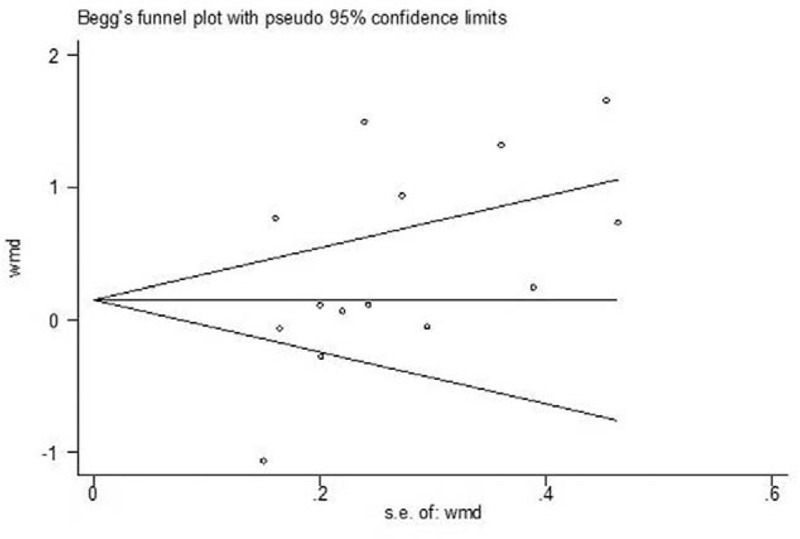
Begg Funnel plot analysis to detect publication bias for complications.

Begg funnel plots and Egger test were used to explore the presence of publication bias for every outcome. Begg funnel plots indicate the effect estimate and confidence interval. If Egger test suggested a distribution of symmetry around the effect evaluations, then the publication bias was likely to be minimal for those studies and outcomes. The funnel plots revealed that none of the outcomes had any significant publication bias (*P* > .05). A funnel plot of the studies included in the primary outcome of complications was created to explore publication bias (Fig. 6).

## 4 Discussion

In this meta-analysis, we found that RPLS and CMPLS showed comparable effectiveness and safety in the treatment of gastric diseases. RPLS is a new, minimally invasive technique that was developed to reduce morbidity, improve aesthetic outcomes, and maintain the optimal results of conventional laparoscopic surgery. This new technique was first described in the 1990s but continued to evolve until 2007.^[32]^ One of the main concerns has always been its safety. To date, many studies have confirmed the technical feasibility and clinical safety of RPLS, including several meta-analyses on RPLS in cholecystectomy,^[33]^ appendectomy,^[34]^ colectomy^[35]^ and inguinal hernioplasty.^[36]^ However, limited data are available on the role of RPLS in gastric diseases. Due to the technical difficulties related to lymph node dissection and reconstruction, this type of surgery has rarely been used for treating gastric diseases.

Several studies have compared RPLS and CMPLS in the treatment of gastric diseases,^[17,18,23,25,29]^ but their sample sizes were small, and they were not individually powered to detect small differences in outcomes. A pooled synthesis of these studies may provide further insight into the safety and comparative effectiveness of RPLS and CMPLS. The pooled studies were heterogeneous due to different RPLS approaches (RPLDG, RP-LATG, or SILS-AGB).

Considering that the operation is performed without any external assistance in RPLS, it is natural that the operative time is usually increased. We also think that RPLS has a longer operative time than CMPLS. In addition, fewer surgical instruments are used in RPLS which makes intraoperative hemostasis difficult. A smaller operating space is available in the peritoneal cavity during single-port laparoscopic surgery which may impact the operation. Thus, RPLS is technically more difficult and involves a longer operative time. The RP-LATG and C-LATG subgroups supported this trend, and pooling these trials revealed an overall significantly longer operating time for RPLS than for CMPLS, which has important implications for both patients and health care providers. Longer surgical procedures mean that patients are exposed to protracted anesthesia, which can increase not only the direct cost of the procedure but also the morbidity and mortality rates.^[37]^ However, it should be noted that the longer operative times for RPLS may partially reflect an initial learning curve; we believe that the longer operative times for RPLS may be overcome with increased experience, standardized training, and meticulous application of the technique. Therefore, careful patient selection is recommended because it could help reduce the slope of the learning curve.

In general, RPLS was developed in an attempt to further minimize the wound access trauma by reducing the number of puncture wounds in the abdominal wall and thereby reduce the bleeding. However, this type of surgery involves a longer operative time, which means more blood loss during the procedure. The RP-LATG vs C-LATG and SILS-AGB vs LAGB subgroups showed similar quantities of blood loss, but the RPLDG vs CLADG subgroup showed a contrasting result, and the pooled analysis confirmed reduced blood loss during RPLS.

The majority of subgroups showed shorter hospital stays after RPLS than after CMPLS, but the RPLDG vs CLADG subgroup showed no significant difference. The pooled analysis also revealed shorter hospital stays after RPLS, which has important implications for both patients and health care providers. The Recovery of patients who underwent RPLS for gastric diseases was faster, which indicates greatly improved patient satisfaction.

Because of the technical difficulties related to lymph node dissection and reconstruction, reduced-port surgery has rarely been used for treating gastric diseases. The patients included in this study underwent gastrectomy with regional lymph node dissection (LND) as described by the Japanese gastric cancer treatment guidelines.^[38]^ Gastric resection with a gross resection margin of ≥2 cm and D1 + LND was primarily performed for cT1N0 gastric carcinoma. If a tumor of ≥cT2 or N+ was suspected during the operation, D2 LND was performed along with total omentectomy. The RP-LATG vs C-LATG subgroups showed no significant difference in the number of dissected lymph nodes, but the other subgroups showed contrary results, and the overall pooled results also reflected this trend.

We found that the complications and conversions were not significantly different between the RPLS and CMPLS groups, perhaps because patients were selected carefully in most of the earlier studies. For example, the characteristics of the patients, such as the age, gender, and preoperative BMI, were similar. RPLS was performed by an experienced surgeon. Ileus, wound infection, and seroma were the most common postoperative complications. However, it should be noted that the follow-up duration in each group was short, preventing a long-term evaluation of the complications. Several studies have shown that the rate of conversion is higher in RPLS. The technical difficulties associated with this technique may also be responsible for the high conversion rate, as crowding over the access port or access site can lead to instrument collisions.^[39]^ This hinders the surgical process, and result in a high rate of conversions to open or conventional laparoscopic surgery. However, the subgroup analysis showed that the rate of conversions was not significantly different between any subgroup; therefore, large-scale, well-designed, multicenter studies are needed to further confirm the results of this study.

Overall, our meta-analysis revealed that although RPLS is more effective and safer than CMPLS, it did not offer a significant advantage over CMPLS in terms of the rate of postoperative complications and conversions.

### 4.1 Limitations

The limitations of our meta-analysis are as follows. First, all involved studies were retrospective analyses, and no RCTs were included; thus, there was a potential cause for bias. More importantly, some of the selected studies had a small number of patients which could also potentially cause bias. Second, postoperative BMI and percent excess weight loss (EWL%) are important indicators for evaluating the effectiveness of the SILS-AGB vs LAGB techniques. Both these were, unfortunately, not proven in our meta-analysis. Only a few of the included studies provided available data on BMI and EWL%. Two studies evaluated the effectiveness of gastric banding surgery via BMI and EWL%; the effectiveness of gastric banding surgery using EWL% was evaluated by 5 studies. However, the data provided by 4 of these studies were not reported as means and standard deviations. Therefore, data from these studies were not available. Furthermore, the duration of follow-up of patients was variable, and the longest follow-up time reported in the original studies was 2 years. The shortest follow-up ended at 3 months, and the patients were discharged from the hospital, which resulted in a lack of uniformity in evaluating the effectiveness of gastric banding surgery. Third, it is well known that all surgical outcomes might be influenced by the individual surgeon's learning curve. However, most studies did not explicitly state whether the surgeons were proficient in the RPLS procedure before the trial began. We believe that some differences between RPLS and CMPLS were overcome with increased experience. In the end, it must be pointed out that there are no RCTs comparing the outcomes between RPLS and CMPLS in the literature to date; thus, the efficacy and safety of RPLS in gastric diseases has not been definitively assessed thus far. Our meta-analysis confirmed that RPLS is a safe and feasible surgical approach and is comparable to CMPLS in these aspects. RPLS offers minimal advantages over CMPLS in terms of a shorter hospital stay and reduced blood loss but does not show any statistically significant difference in the rates of complications and conversions. If the results of RCTs prove that RPLS ensures a lower complication rate and better functional results than CMPLS does, the cost-effectiveness of RPLS may be confirmed.

## 5 Conclusions

The effectiveness and safety of RPLS in the treatment of gastric diseases were comparable to those of CMPLS in our meta-analysis. Based on current evidence, we believe that the RPLS is an efficacious surgical alternative to CMPLS in the treatment of gastric diseases because of the shorter hospital stay and reduced blood loss. However, large-scale, well-designed, multicenter studies are needed to further confirm the results of this study.

## Author contributions

**Conceptualization:** Jun Zhou.

**Data curation:** Xu Yang, Zhaoting Bu, Maoqin He, Yue Lin.

**Formal analysis:** Zhaoting Bu, Maoqin He, Yue Lin, Kaibing Liu.

**Investigation:** Da Chen, Kaibing Liu.

**Methodology:** Da Chen.

**Software:** Xu Yang.

**Supervision:** Jun Zhou, Yuting Jiang.

**Writing – original draft:** Xu Yang, Maoqin He, Yuting Jiang.

**Writing – review & editing:** Jun Zhou, Zhaoting Bu, Yue Lin, Yuting Jiang.
